# Molecular Epidemiology of SARS-CoV-2 in Tunisia (North Africa) through Several Successive Waves of COVID-19

**DOI:** 10.3390/v14030624

**Published:** 2022-03-17

**Authors:** Anissa Chouikha, Wasfi Fares, Asma Laamari, Sondes Haddad-Boubaker, Zeineb Belaiba, Kais Ghedira, Wafa Kammoun Rebai, Kaouther Ayouni, Marwa Khedhiri, Samar Ben Halima, Henda Krichen, Henda Touzi, Imen Ben Dhifallah, Fatma Z. Guerfali, Chiraz Atri, Saifeddine Azouz, Oussema Khamessi, Monia Ardhaoui, Mouna Safer, Nissaf Ben Alaya, Ikram Guizani, Rym Kefi, Mariem Gdoura, Henda Triki

**Affiliations:** 1 Reasearch Laboratory “Virus, Vectors and Hosts: One Health Approach and Technological Innovation for a Better Health”, LR20IPT02, Institut Pasteur de Tunis, Université Tunis El Manar, Tunis 1002, Tunisia; wasfi.feres2020@gmail.com (W.F.); alamari445@gmail.com (A.L.); sondeshaddadboubaker@gmail.com (S.H.-B.); zeineb.belaiba114@gmail.com (Z.B.); kaouther.ayouni@gmail.com (K.A.); marwakehidiri@gmail.com (M.K.); touzihenda@yahoo.fr (H.T.); bendhifallahimene@gmail.com (I.B.D.); mariemgdoura@gmail.com (M.G.); henda.triki@pasteur.tn (H.T.); 2 Laboratory of Clinical Virology, WHO Reference Laboratory for Poliomyelitis and Measles in the Eastern Mediterranean Region, Institut Pasteur de Tunis, Université Tunis El Manar, Tunis 1002, Tunisia; bh_samar80@yahoo.fr (S.B.H.); henda.krichen@gmail.com (H.K.); 3 Clinical Investigation Center (CIC), Institut Pasteur de Tunis, Université Tunis El Manar, Tunis 1002, Tunisia; ardhaouimonia@gmail.com (M.A.); iguizani@yahoo.com (I.G.); 4 Laboratory of Bioinformatics, Biomathematics and Biostatistics (BIMS), Institut Pasteur de Tunis, Université Tunis El Manar, Tunis 1002, Tunisia; ghedirakais@gmail.com; 5 Laboratory of Biomedical Genomics and Oncogenetics (LR16IPT05), Institut Pasteur de Tunis, Université Tunis El Manar, Tunis 1068, Tunisia; kammoun.wafa@gmail.com (W.K.R.); rym.kefi@pasteur.utm.tn (R.K.); 6 Laboratory of Transmission, Control and Immunobiology of Infections (LTCII) (LR16IPT02), Institut Pasteur de Tunis, Université Tunis El Manar, Tunis 1068, Tunisia; fatma.guerfali@pasteur.utm.tn (F.Z.G.); chiraz.atri@pasteur.tn (C.A.); 7 Genomics Plateform, Institut Pasteur de Tunis, Université Tunis El Manar, Tunis 1068, Tunisia; saifeddine.azouz@pasteur.tn; 8 Laboratoire de Venins et Biomolécules Thérapeutiques (LR16IPT08), Institut Pasteur de Tunis, Université Tunis El Manar, Tunis 1068, Tunisia; oussama.khamassi@pasteur.tn; 9 Laboratory of Molecular Epidemiology & Experimental Pathology (LR16IPT04), Institut Pasteur de Tunis, Université Tunis El Manar, Tunis 1068, Tunisia; 10 National Observatory of New and Emergent Diseases, Tunis 1002, Tunisia; safermouna@gmail.com (M.S.); nissaf.bouafif@rns.tn (N.B.A.)

**Keywords:** SARS-CoV-2, Tunisia, variant of concern, variant of interest, variants, next-generation sequencing, molecular characterization, COVID-19, partial sequencing, spike gene, next-generation sequencing

## Abstract

Documenting the circulation dynamics of SARS-CoV-2 variants in different regions of the world is crucial for monitoring virus transmission worldwide and contributing to global efforts towards combating the pandemic. Tunisia has experienced several waves of COVID-19 with a significant number of infections and deaths. The present study provides genetic information on the different lineages of SARS-CoV-2 that circulated in Tunisia over 17 months. Lineages were assigned for 1359 samples using whole-genome sequencing, partial S gene sequencing and variant-specific real-time RT-PCR tests. Forty-eight different lineages of SARS-CoV-2 were identified, including variants of concern (VOCs), variants of interest (VOIs) and variants under monitoring (VUMs), particularly Alpha, Beta, Delta, A.27, Zeta and Eta. The first wave, limited to imported and import-related cases, was characterized by a small number of positive samples and lineages. During the second wave, a large number of lineages were detected; the third wave was marked by the predominance of the Alpha VOC, and the fourth wave was characterized by the predominance of the Delta VOC. This study adds new genomic data to the global context of COVID-19, particularly from the North African region, and highlights the importance of the timely molecular characterization of circulating strains.

## 1. Introduction

Severe acute respiratory syndrome coronavirus 2 (SARS-CoV-2), the cause of coronavirus disease 2019 (COVID-19), was first detected in December 2019 in the city of Wuhan in Hubei Province of China, in a patient with acute pneumonia [1,2,3]. On 11 March 2020, the World Health Organization (WHO) characterized COVID-19 as a pandemic. As of 06 March 2022, more than 440 million confirmed cases and more than 6 million deaths have been reported worldwide [4]. The first whole-genome sequence of SARS-CoV-2 was published on 5 January 2020 [2], and since then, the analysis of viral sequences worldwide has been continuous, with more than 2.5 million complete genomes currently available in public databases, such as the GISAID platform [5]. Molecular analysis has shown significant genetic variability of the SARS-CoV-2 virus due to the accumulation of mutations over time. Most of these changes have little to no impact, but some mutations have an impact on viral properties and lead to an increase in virus transmissibility, more severe infection, a potential reduction in vaccine or immune effectiveness and/or escape from molecular diagnosis. Variants that have acquired at least one of these characteristics are named variants of concern (VOCs) and require special monitoring. In addition, other variants are classified as variants of interest (VOIs), variants under monitoring (VUMs) or variants under investigation (VUIs) [6,7,8].

By July 2021, four major variants of concern (VOCs) had been described that led to increased surveillance efforts worldwide [9]. The Alpha variant, B.1.1.7 lineage (20I/501Y.V1, VOC-202012/01), also known as the UK variant, has an unusually high number of mutations and is more transmissible than the wild-type virus [10]. The Beta variant, B.1.351 lineage (501.V2, 20H/501Y.V2, VOC-202012/02), first detected and reported in South Africa in early October 2020, shares several mutations with B.1.1.7 and reduces vaccine effectiveness to some extent [11,12]. The Gamma variant, P.1 lineage (20J/501Y.V3, VOC-202101/02), emerged in December 2020 in Brazil [13]; it has 10 mutations in the spike protein that may affect its ability to be recognized by antibodies [14,15]. The Delta variant (B.1.617.2, AY.1 and AY.2 VOC-21APR-02) was first described in India and then widely spread all over the world [11,12]. This variant has eight mutations in the spike protein and is characterized by increased transmissibility in comparison with the Alpha variant [11,12]. In November 2021, a new VOC designated the Omicron variant (B.1.1.529) was first described in Botswana and in South Africa. This new variant has rapidly spread all over the world and is presently the most frequently detected worldwide [16,17]. This variant exhibits a large number of mutations, among which more than 30 are in the spike protein. The substitution mutations Q493R, N501Y, S371L, S373P, S375F, Q498R and T478K in the spike protein are suggested to result in increased transmission due to a higher affinity for human angiotensin-converting enzyme 2 (ACE2). Moreover, an increased risk of reinfection is observed with this variant as compared to other VOCs [16,17]. Therefore, the molecular monitoring of circulating strains is crucial for the timely identification of the emergence of novel SARS-CoV-2 variants.

In Tunisia, the first cases of COVID-19-positive patients were reported in early March 2020, all of which were imported cases. By June 2020, 1155 cases had been reported, which were imported or import-related: persons coming from foreign countries and testing positive at arrival to Tunisia or persons who had been in contact with imported cases detected through contact tracing. After drastic decisions were taken by the Tunisian government, such as global lockdown, early detection of imported and local cases, quarantining of confirmed/suspected cases and border closures, case numbers reached zero between 4 and 12 June 2020 [18]. The second wave started in July 2020 after the borders reopened and a notable relaxation in compliance with preventive measures by the general population. After a small decrease in disease incidence in February 2021, the country experienced a third wave of COVID-19 with the introduction of the Alpha variant in March 2021 and then fourth and fifth waves after the introduction of Delta and Omicron in May and December 2021, respectively.

The aim of the present study is to provide genetic information on the different lineages of SARS-CoV-2 that circulated in Tunisia during a period of 17 months (March 2020–July 2021) covering different waves.

## 2. Materials and Methods

### 2.1. Sample Collection

This study is based on nasopharyngeal samples tested in the Laboratory of Clinical Virology of Pasteur Institute of Tunis, mandated to perform COVID-19 diagnosis by the Tunisian Ministry of Health (MoH) as part of the national program of surveillance of SARS-COV-2. From March 2020 to 31 July 2021, 125,456 nasopharyngeal swab samples were referred from public and private health institutions, of which 28,517 (22.7%) were confirmed SARS-CoV-2-positive by real-time RT-PCR. Variant detection and lineage characterization were performed on a randomly selected subset of samples (N = 1359) covering the whole 17-month period and representing the 24 governorates of Tunisia.

Three different techniques were used for the timely determination of the lineages circulating: whole-genome sequencing, partial S gene sequencing and real-time RT-PCR. The methods used for SARS-CoV-2 lineage and sub-lineage determination by month of sample collection are shown in Supplementary Material S1.

### 2.2. Whole-Genome Sequencing

Whole-genome sequencing was performed for 601 specimens with Ct values less than 30 that were collected throughout the whole 17-month study period. Of the 601 specimens, 10 were sequenced using MinION technology, and n = 591 were sequenced using Illumina technology as follows: RNA was extracted from a 140 μL nasopharyngeal sample with the Qiamp viral RNA mini kit (Qiagen, Hilden, Germany) according to the manufacturer’s instructions. Genomes were generated by an amplicon-based approach using the United States Food and Drug Administration (US FDA)-approved Illumina COVIDSeq kit (San Diego, CA, USA) [19], a modified version of the ARTIC protocol. The extracted RNAs were reverse transcribed to single-strand cDNA using Superscript IV Reverse Transcriptase (Invitrogen™, Waltham, MA, USA) as per the manufacturer’s instructions. SARS-CoV-2 PCR with two specific primer pools combined with proven Illumina sequencing technology allowed us to obtain 98 tiled amplicons covering the whole genome. For each sample, PCR products were combined, and libraries were prepared using the Illumina-Nextera DNA UD Indexes as per the manufacturer’s instructions. Libraries were purified with AMPure XP magnetic beads (Beckman Coulter, Brea, CA, USA), and concentration was measured by Qubit dsDNA HS Assay kit (Thermo Fisher Scientific, Waltham, MA, USA). Library pool validation and mean fragment size were determined by Bioanalyzer 2100 (Agilent, Santa Clara, CA, USA) as per the manufacturer’s instructions. The 400 bp library pool was diluted to 4 nM. Libraries were pooled, denatured, diluted to 1.4 pM and sequenced on a NextSeq550 instrument with a V2.5 High Output Cartridge 2 × 150 bp run kit (Illumina, San Diego, CA, USA). Raw sequence data were processed using fastqc version 0.11.9 for quality control (https://www.bioinformatics.babraham.ac.uk/projects/fastqc/ (accessed on 30 August 2021). Low-quality reads and adapters were filtered using trimmomatic version 0.39 with a Phred quality score of 30 as the threshold.

### 2.3. Genome Assembly

For the sequences generated by ONT MinION technology (Oxford Nanopore Technology, Oxford, UK), the depth ranged between 564× and 2.263×, and coverage ranged between 22,776 and 29,126. A modified ARTIC network pipeline v1.0.0 was used to generate consensus genomes with a reference-based genome assembly pipeline and read length filtering against the Wuhan-Hu-1 (RefSeq accession: NC_045512.2), and variants were polished using nanopolish v0.13.2, medaka v0.11.5 and samtools 1.9. For the sequences generated using Illumina Technology, the genomes were assembled using the EDGE COVID-19 pipeline, which is based on the fully open-source EDGE Bioinformatics software [20]. FaQCs was used to control the quality of the reads [21]. Low-quality regions of reads were trimmed and filtered if the reads failed a quality threshold of 20 or minimum length of 50 bp. The reads that passed the QC process were then aligned to the original Wuhan-Hu-1 complete reference genome (RefSeq accession: NC_045512.2) using BWA-mem [22]. Various parameters were set to default values: (i) minimum depth coverage (5×) to support a variant site, (ii) alternate base threshold (0.5) to support an alternative for a change in the consensus base, (iii) indel threshold (0.5) to support an INDEL for a change in the consensus base and (iv) minimum mapping quality [23].

### 2.4. Variant Detection by Partial Sequencing of the S Gene

Amplification by standard PCR and partial sequencing using Sanger technology was used for 358 samples collected from February to July 2021, as described previously [24]. The 648-nucleotide-long S gene sequence encodes for the 477 to 693 amino acid residue region of the S protein. It includes key positions and allows the detection of the most important mutations characterizing most VOCs, VOIs and VUMs.

### 2.5. Variant Detection by Real-Time RT-PCR

The commercial kit SNPsig^®^ real-time PCR SARS-CoV-2 mutation detection/allelic discrimination kit (Primerdesign Ltd., Southampton, UK), which allows the detection of the Alpha, Beta and Gamma variants, was used for 400 samples collected from March to June 2021, during the high-transmission period of the Alpha variant. The test is based on the search for the first step of the N501Y substitution, which is common to the Alpha, Beta and Gamma variants. Discrimination between the three variants is then performed for all samples harboring the N501Y substitution using primers and probes specific to each variant.

### 2.6. Clade and Lineage Assignment

The Fasta format of whole genome sequences was used for clade and lineage assignment using online tools: Nextclade [10] and Pangolin (version 3.1.16, lineages version 2021-11-25) [25].

### 2.7. Phylogenetic Analysis 

Multisequence alignment was performed with MAFFT using default parameters (https://mafft.cbrc.jp/alignment/server/ (accessed on 10 March 2022)) [26]. The resulting alignment was used to build a maximum likelihood phylogenetic tree using IQ-TREE online web server using an automatic substitution model supported by 1000 bootstrap replicates (http://iqtree.cibiv.univie.ac.at/ (accessed on 10 March 2022)) [27]. The phylogenetic tree was then visualized using Figtree software (http://tree.bio.ed.ac.uk/software/figtree/ (accessed on 10 March 2022)). Bootstrap values less than 60 are not shown in the tree. A Nextclade tree was also constructed using the online tool (https://clades.nextstrain.org/ (accessed on 10 March 2022)). The tree was then downloaded in JSON format and visualized using the Auspice v2.34.0 online tool (https://auspice.us/ (accessed on 10 March 2022)).

## 3. Results

**Figure 1:**
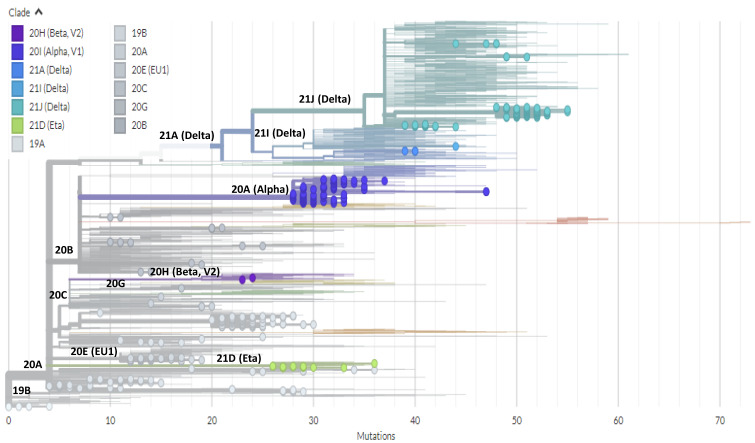
Phylogenetic tree built using Nextclade online software (https://clades.nextstrain.org/tree (accessed on 10 March 2022))) and visualized using Auspice online tool (https://auspice.us/ (accessed on 10 March 2022) based on n = 463 severe acute respiratory syndrome coronavirus 2 (SARS-CoV-2) cases detected in Tunisia between March 2020 and July 2021. The circles represent the Tunisian sequences in comparison with published sequences from all over the world. Here, Nextclade lineages are broken down according to the indicated color code.

**Figure 2:**
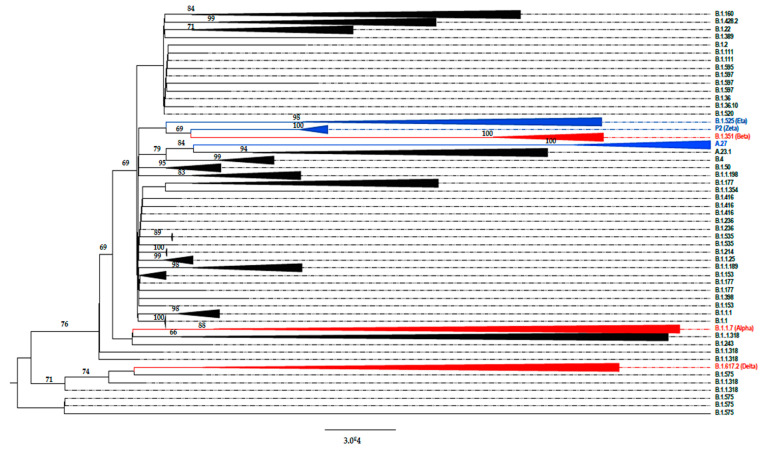
Phylogenetic tree built using IQ-tree and visualized by Figtree based on n = 601 severe acute respiratory syndrome coronavirus 2 (SARS-CoV-2) whole genomes detected in Tunisia between March 2020 and July 2021. The triangles in red correspond to variant of concern (VOC) sequences, variant of interest (VOI) sequences are shown in blue, and other lineages that circulated in Tunisia during the study period are represented in gray. The numbers on branches represent the bootstrap support values.

In the present study, lineages could be successfully assigned to the 1359 SARS-CoV-2 samples using one of the three described methods. Overall, between March 2020 and 31 July 2021, a very high viral diversity was observed, with the identification of 48 different lineages (Table 1). According to the Pangolin lineage classification, the overwhelming majority of lineages detected (97.8%) belonged to B clade, with the low circulation of A (1.8%) and P (0.4%) clades (https://pangolin.cog-uk.io/ (accessed on 10 March 2022)). According to the Nextclade classification [10], 14 different clades were detected in Tunisia: 19A, 19B, 20A-E, 20G-I, 21A, 21D, 21I and 21J. Figure 1 shows the phylogenetic tree built using the Nextclade online tool (https://clades.nextstrain.org/ (accessed on 10 March 2022)). Figure 2 shows the phylogenetic tree based on Pangolin lineage classification and Table 1 illustrates the correspondence between the WHO, Pangolin lineage and Nextclade assignments according to the months of sample collection.

**Figure 3:**
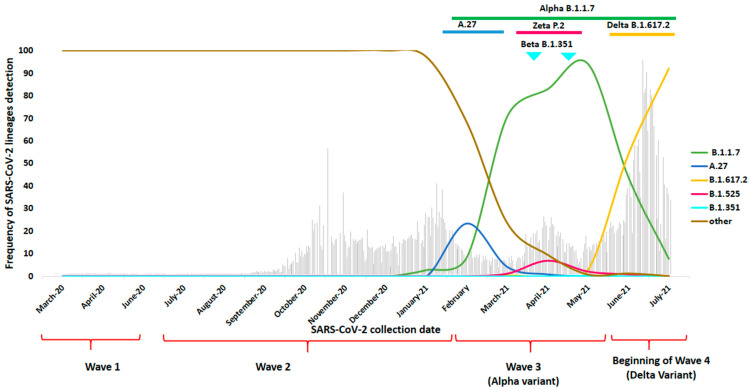
Emergence of variants of concern (VOCs) and variants of interest (VOIs) in Tunisia between March 2020 and July 2021. In the background, is the number of positive cases detected in Tunisia according to the statistics published in the World Health Organization (WHO) website [18].

Table 1 and Figure 2 and Figure 3 report the different SARS-CoV-2 lineages detected during the four different waves of the disease in Tunisia during the 17-month study period.

The first wave ranged from March to June 2020 and was characterized by small numbers of circulating lineages (n = 7), namely, B.1.153; B.1.36, B.1.36.10; B.1.398, B.1.214, B.520 and B.4, corresponding to the 19A and 20A Nextclade classifications.

The second wave ranged from July 2020 to January 2021; it was characterized by a higher genetic diversity with the circulation of at least 20 different lineages, namely: B.1.1.50, B.1.597, B.1.1.1, B.1.22, B.1.428.2, B.1.1.25, B.1.1.198, B.1.1.189, B.1.1.354, B.1.177, B.1.160, B.1.111, B.1.389, B.1.575, B.1.473, B.1.595, B.1.623, B.1.236, B.1.535, B.1.533 and B.1.416 (20A to 20E according to the Nextclade classification) (Table 1).

The third wave ranged from February to May 2021 and was characterized by the emergence of variants of concern (VOCs) and variants of interest and/or under monitoring. Indeed, the A.27 lineage, considered at that time to be a variant of interest, was first detected in Tunisia in February 2021, and the last isolate was identified in April 2021 (Table 1). The A.27 lineage is recognized by the following substitutions in the S gene: L18F, L452R, N501Y, A653V, H655Y, D796Y and G1219V, and was first detected in the present study using partial S gene sequencing. Starting from March 2021, the Alpha B.1.1.7 (UK) VOC, defined by multiple spike protein mutations, namely, deletions at 69–70 and 144, N501Y, A570D, D614G, P681H, T716I, S982A and D1118H, displaced the previous A.27 lineages and rapidly became the predominant variant detected in the country. Another VOC, the Beta (B.1.351) variant, known by the following S protein mutations: K417N, E484K, N501Y, D614G and A701V, was also detected in two cases, one in April and one in May 2021 during the third wave. Wave 3 was also characterized by the detection of a few cases of the Zeta (P.2) variant, the alias of B.1.1.28.2, also known as the Brazilian lineage, as well as the Eta (B.1.525) variant, known by the E484K substitution of interest and the del69–70 deletion. Zeta and Eta were, at that time, assigned as VUMs (variants under monitoring) (Table 1, Figure 3). Other sporadic lineages were also detected during the third wave: B.1.533, B.1.416, A.23.1, B.1.243, B.1.415, B.1.160, B.1.1.178, B.1.620, B.1.1.318 and B.1.2 (Table 1).

During the beginning of the fourth wave (June–July 2021), the overwhelming majority of detected lineages was the Delta B.1.617.2 variant, known by the following mutations in the S protein: L452R, T478K, D614G and P681R. The Delta VOC rapidly displaced the Alpha VOC, which circulated with very few cases together with Eta, B.1.367 and B.1.575 lineages (Table 1, Figure 2).

It is also important to note that some lineages, such as B.1.177 and B.1.160, circulated for a long period covering waves 2 and 3, extending from September 2020 to April–May 2021.

## 4. Discussion

In this retrospective observational study, we describe the SARS-CoV-2 lineages that circulated in Tunisia for 17 months after its first introduction to the country in March 2020. Significant genetic diversity of SARS-CoV-2 was observed with the circulation of many SARS-CoV-2 lineages during four different waves that the country experienced up to July 2021. This could be explained by different virus importations. Several factors could favor the multiple introductions of these different viral lineages to Tunisia and their rapid spread. First, Tunisia, a small country with an area of 163,610 km^2^ and a population of approximately 12 million, has a strategic geographic location, which makes it a junction point between the Arab world, Africa and Europe. Furthermore, it is known for its history of economic and cultural transactions, particularly with European and neighboring countries. In addition, Tunisia was experiencing an economic and political crisis that prevented total lockdown for long periods, and the introduction of the anti-SARS-CoV-2 vaccine to the population was relatively late.

The succession of the different waves observed in Tunisia is similar to the global picture of COVID-19 infection (https://covid19.who.int/ (accessed on 30 August 2021)). In addition, the same picture of the circulation of several viral lineages has been reported in several countries around the world, such as the Czech Republic, Cyprus, the UK, Russia, South Africa and countries from the Middle East and North Africa (MENA) region [28,29,30,31,32,33]. The exchange between countries has played a crucial role in the importation of new lineages and their rapid spread across countries.

This phenomenon is characteristic of airborne viruses. Countries that have succeeded in stopping their transmission are those that have applied drastic measures, such as China and South Korea, or other countries that were able to contain the virus during the first phases of the pandemic with full containment, border closures and the minimization of any contact, even between non-infected people [34,35,36,37,38].

In Tunisia, the first wave began with the introduction of the virus in March 2020; the drastic decisions taken by the government have effectively controlled the pandemic with a very low rate of infected persons that reached zero cases in June 2020. During this first wave, full compliance with sanitary measures by the population and the adequate decisions taken by decision-makers made it possible to contain the disease. At that time, the number of patients was not significant, especially those with severe disease requiring resuscitation and oxygen beds. On 27 June 2020, it was decided to reopen the borders. Countries were classified into three different categories depending on their epidemiological situation, and passengers coming from countries with low endemicity (green zones) were not required to self-isolate. This led to the re-emergence of the virus since some people coming from green zones were positive and reintroduced the virus in the country. Moreover, the summer period was marked by the return of emigrants and summer festivities, together with non-compliance with sanitary measures by the general population. This led to the outbreak of a second wave of COVID-19 with local transmission and two peaks coinciding with the start of the school year in September/October and the festivities celebrating the New Year in December 2020/January 2021. After a small decrease in disease incidence in February 2021, the country experienced a third wave of COVID-19 with the introduction of the Alpha VOC and A.27 VOI.

The A.27 lineage was first detected in February 2021 concomitantly with its widespread emergence in France, a country with which Tunisia has important cultural and economic exchanges. At that time, the A.27 lineage, also named the Henry Mondor variant, was considered to be a variant of interest or variant under monitoring since it caused many grouped cases in France and harbors mutations described in VOCs [39]. The A.27 lineage shares substitutions with VOCs such as L18F, L452R and N501Y, which have been suggested to result in immune escape and higher transmissibility [40]. Currently, the A.27 lineage is not considered a VOI (https://www.who.int/en/activities/tracking-SARS-CoV-2-variants/ (accessed on 16 March 2022). Thereafter, it was quickly displaced by the Alpha B.1.1.7 VOC. The Alpha VOC, first detected in the United Kingdom in late 2020, is defined by an N501Y amino acid substitution in the spike protein that increases its transmissibility. The Alpha VOC had become the dominant global variant by early 2021 [5,33,40,41]. In Tunisia, it was first detected in January 2021 and rapidly became the dominant lineage. During this third wave, real-time PCR testing detecting the three VOCs detected around the world at that time (Alpha, Beta and Gamma) was used and allowed rapid screening for this variant.

Other variants that have raised interest at the international level were also detected in our series, including the Beta variant (B.1.351), also known as 20H or variant of concern 501Y.V2. Beta was first described in South Africa and reported by Tegally et al. [11], and it was then detected in several countries on all continents, with the highest period of transmission between October 2020 and September 2021. In our series, it was detected in two travelers upon their arrival to Tunisia in April and May 2021; its early detection and the timely decisions taken contained this variant, hampering its spread in the country. The A.23.1 lineage was also detected. This lineage emerged in September 2020 and has several mutations with potential biological concern, including the 681R substitution, although it has not been classified as a VOC or VOI [42]. It was reported in several countries: 18 in Europe, 12 in Asia and 16 in sub-Saharan Africa, the USA, Canada and Australia. To our knowledge, it is reported herein for the first time in North Africa. The Eta (B.1.525) and Zeta (P2 or B.1.1.28) variants were also detected.

Moreover, other lineages circulated for long periods, such as B.1.160 and B.1.177, which took an important place in the lineage landscape circulating in Tunisia. These lineages circulated from September 2020 until mid-2021 without any impact on the overall epidemiological situation. B.1.160, known as 20A/EU2, is one of the main variants first reported in Europe [43]. The B.1.177 lineage, mostly detected in Europe, was first detected in early 2020 and is currently classified into more than 80 sub-lineages [42]. Further molecular characterization of a higher number of viruses will be of great interest to better characterize these two lineages.

In May 2021, the Delta variant, characterized by L452R and P681H amino acid substitutions in the spike protein, was detected in the country and rapidly displaced the Alpha variant, becoming the dominant variant in June–July 2021. This VOC, first detected in India in early 2021, became the most frequently detected variant in many countries [5,44,45]. Indeed, it was demonstrated that the Delta variant emerged faster than the Alpha variant and dominated the variant landscape worldwide. In the present study, the emergence of the Delta variant defined the fourth wave of SARS-CoV-2 infection in the country and participated in the resurgence of SARS-CoV-2 cases. The circulation of the Delta variant coincided with high transmissibility and with a large number of severe disease cases. In fact, infection with the Delta variant is characterized by the generation of an average of 6 times more viral RNA copies per milliliter than Alpha infections [12]. In Tunisia, the detection of the Delta variant decreased from August to December 2021 (data not shown).

The disease incidence increased again with the introduction of Omicron, which was first detected in early December and caused a new wave with a much higher transmission rate.

## 5. Conclusions

This study describes the Tunisian experience in the molecular surveillance of SARS-CoV2. The generated genomic data contribute to the enrichment of the globally published data on SARS-CoV-2 circulation, particularly in North Africa. It highlights the efforts that have been made for rapid and efficient detection of variants despite the main limitation of the unavailability of onsite NGS technology. Lineage and sub-lineage assignments were performed using multiple methods, including whole-genome sequencing, partial sequencing of the S gene and screening for VOCs by real-time PCR. Although partial sequencing and VOC detection by real-time PCR do not allow complete characterization of lineages and sub-lineages, they were of great help in promptly identifying the introduction of the main VOCs, especially Alpha and Delta. Our study also points to three important measures that should be considered to prevent the emergence of new waves and new virus(es) introduction(s): (1) continuous respect of strict prevention measures without becoming complacent; (2) timely identification of new variants via clear procedures of genomic surveillance and (3) the strengthening of vaccination by raising awareness in the general population.

## Figures and Tables

**Table 1:** Severe acute respiratory syndrome coronavirus 2 (SARS-CoV-2) lineage distribution in Tunisia from March 2020 to July 2021 according to month of sample collection. Variants of concern (VOCs) and variants of interest (VOIs) are shown in red and in blue, respectively.

					Wave 1			Wave 2							Wave 3			Begining of Wave 4			
WHO	GISAID Clade	Nextstrain Clade	Methodolgy	Pango Lineage	Mar-20	Apr-20	Jun-20	Jul-20	Aug-20	Sep-20	Oct-20	Nov-20	Dec-20	Jan-21	Feb-21	Mar-21	Apr-21	May-21	Jun-21	Jul-21	Total
			**WGS**	**B.1.153**	**1**	**1**															**2**
			**WGS**	B.1.1 *	**1**	**4**			**5**	**6**	**1**	**5**		**2**	**1**				**1**		**26**
		**20A**	**WGS**	**B.1.36.10**	**1**																**1**
			**WGS**	B.1 *	**12**	**9**	**2**	**1**	**7**	**4**	**4**	**13**	**1**	**12**	**5**	**7**	**8**		**7**		**92**
			**WGS**	**B.1.520**		**1**															**1**
		**19A**	**WGS**	**B.4**		**3**															**3**
		**20A**	**WGS**	**B.1.36**			**1**														**1**
		**20A**	**WGS**	**B.1.398**			**1**														**1**
		**20A**	**WGS**	**B.1.214**			**2**														**2**
		**20B**	**WGS**	**B.1.1.50**				**6**				**1**									**7**
		**20C**	**WGS**	**B.1.597**					**1**		**1**										**2**
		**20D**	**WGS**	**B.1.1.1**					**1**			**1**									**2**
		**20A**	**WGS**	**B.1.22**					**2**	**1**	**2**										**5**
		**20C**	**WGS**	**B.1.428.2**					**2**	**2**	**6**	**3**	**2**		**1**						**16**
		**20B**	**WGS**	**B.1.1.25**					**2**												**2**
		**20B**	**WGS**	**B.1.1.198**						**1**	**2**	**1**		**1**		**1**					**6**
		**20B**	**WGS**	**B.1.1.189**						**1**	**3**										**4**
			**WGS**	**B.1.1.354**						**1**											**1**
		**20E**	**WGS**	**B.1.177**						**2**	**7**	**6**	**2**	**6**	**4**	**7**	**4**				**38**
		**20A**	**WGS**	**B.1.160**						**3**	**15**	**15**	**3**	**11**	**13**	**26**	**1**	**1**			**88**
			**WGS**	**B.1.111**							**1**				**1**						**2**
		**20A**	**WGS**	**B.1.389**							**1**										**1**
			**WGS**	**B.1.575**								**1**							**2**	**1**	**4**
		**20A**	**WGS**	**B.1.473**								**1**									**1**
		**20C**	**WGS**	**B.1.595**								**1**									**1**
			**WGS**	**B.1.623**								**1**									**1**
		**20A**	**WGS**	**B.1.236**									**1**			**1**					**2**
			**WGS**	**B.1.535**									**1**								**1**
		**20A**	**WGS**	**B.1.533**										**1**	**1**						**2**
**Alpha**	**GRY**	**20I**	**WGS**	**B.1.1.7**											**1**	**25**	**79**	**11**	**6**	**1**	**713**
			**PS/qRT-PCR**											**1**	**3**	**113**	**79**	**277**	**112**	**5**	
		**20A**	**WGS**	**B.1.416**										**1**					**1**		**2**
			**WGS**	B *										**1**							**1**
		**20A**	**WGS**	**B.1.243**											**1**						**1**
**Zeta**		**20B**	**WGS**	**P.2**											**4**	**1**					**5**
		**19B**	**WGS**	**A.27**											**8**	**6**	**3**				**23**
			**PS**												**3**	**2**	**1**				
		**19B**	**WGS**	**A.23.1**												**1**	**1**				**2**
			**WGS**	**B.1.415.1**												**1**					**1**
**Eta**	**G/484K.V3**	**21D**	**WGS**	**B.1.525**												**2**	**6**	**1**	**1**		**20**
			**PS**														**3**	**5**	**2**		
		**20A**	**WGS**	**B.1.160.14**												**2**					**2**
		**20A**	**WGS**	**B.1.160.28**												**2**					**2**
**Beta**	**GH/501Y.V2**	**20H**	**WGS**	**B.1.351**													**1**				**2**
			**PS**															**1**			
			**WGS**	**B.1.1.178**													**1**				**1**
	**G**	**20A/S:126A**	**WGS**	**B.1.620**													**2**	**1**			**3**
		**20E**	**WGS**	**B.1.1.318**													**2**	**6**	**1**		**9**
		**20G**	**WGS**	**B.1.2**													**2**				**2**
**Delta**	**G/478K.V1**	**21A,21I,21J**	**WGS**	**B.1.617.2**														**4**	**84**	**16**	**253**
			**PS**															**6**	**92**	**51**	
		**20C/S:80Y**	**WGS**	**B.1.367**															**1**		**1**
			**WGS**	**B.1.629**																**1**	**1**
				**Total**	**15**	**18**	**6**	**7**	**20**	**21**	**43**	**49**	**10**	**36**	**46**	**197**	**193**	**313**	**310**	**74**	**1359**

* Lineages without any sub-lineage assignment, such as B, B.1 and B.1.1, were observed in n = 1, n = 92 and n = 26 cases, respectively. This was due to the quality of sequences generated, which did not allow proper assignment to a sub-lineage. PS = partial sequencing in the S gene; qRT-PCR = real-time PCR; and WGS = whole-genome sequencing.

## Data Availability

The data that support the findings of this study are available from the corresponding author upon reasonable request.

## Supplementary Materials

The following supporting information can be downloaded at: https://www.mdpi.com/article/10.3390/v14030624/s1, Supplementary Material S1. Methods used for SARS-CoV-2 Lineages and sub-lineages determination by month of sample collection.
