# Supplementary material for: Molecular Epidemiology of SARS-CoV-2 in Tunisia (North Africa) through Several Successive Waves of COVID-19
Source: Viruses. 2022 Mar 17;14(3):624. doi: 10.3390/v14030624 (PMC8956073; doi:10.3390/v14030624)

Supplementary Material S1. Methods used for SARS-CoV-2 Lineages and sub-lineages determination by month of sample collection

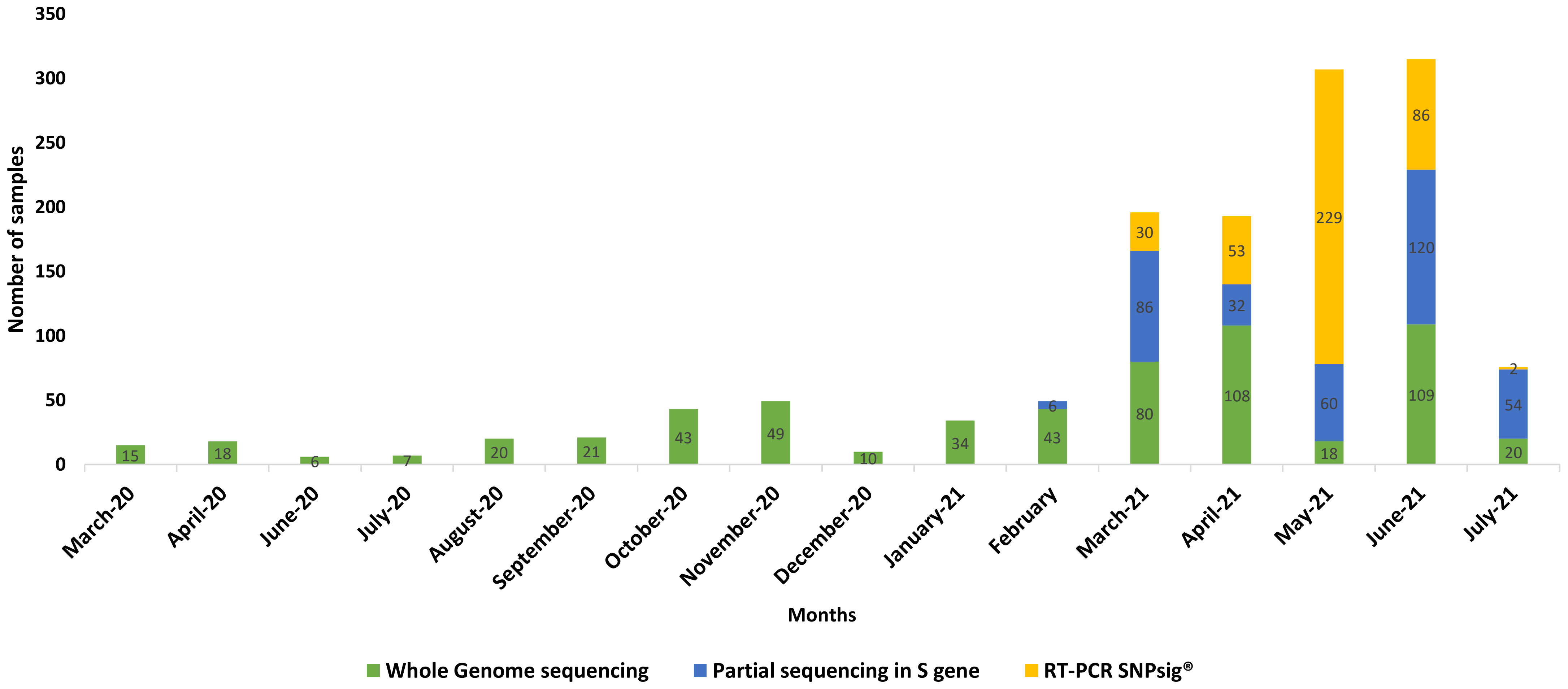

Supplement: Supplementary file 1 [file viruses-14-00624-s001.zip › viruses-1577281-supplementary.pdf]
